# Bacteria-Induced Acute Inflammation Does Not Reduce the Long-Term Reconstitution Capacity of Bone Marrow Hematopoietic Stem Cells

**DOI:** 10.3389/fimmu.2020.00626

**Published:** 2020-04-16

**Authors:** Xiaoyu Zhang, Kutay Karatepe, Direkrit Chiewchengchol, Haiyan Zhu, Rongxia Guo, Peng Liu, Hongbo Yu, Qian Ren, Xiao Luo, Tao Cheng, Fengxia Ma, Yuanfu Xu, Mingzhe Han, Hongbo R. Luo

**Affiliations:** ^1^Department of Pathology, Harvard Stem Cell Institute (HSCI), Harvard Medical School, Boston, MA, United States; ^2^Department of Lab Medicine, The Stem Cell Program, Children's Hospital Boston, Boston, MA, United States; ^3^Dana-Farber/Harvard Cancer Center, Boston, MA, United States; ^4^The State Key Laboratory of Experimental Hematology, Institute of Hematology and Blood Diseases Hospital, Chinese Academy of Medical Sciences and Peking Union Medical College, Tianjin, China; ^5^Department of Pathology and Laboratory Medicine, VA Boston Healthcare System, West Roxbury, MA, United States; ^6^Department of Hematopoietic Stem Cell Transplantation, Institute of Hematology and Blood Diseases Hospital, Chinese Academy of Medical Sciences and Peking Union Medical College, Tianjin, China

**Keywords:** hematopoietic stem cells, acute infection, inflammation, long-term reconstitution, self-renewal

## Abstract

Pathogen-initiated chronic inflammation or autoimmune diseases accelerate proliferation and promote differentiation of hematopoietic stem cells (HSCs) but simultaneously reduce reconstitution capacity. Nevertheless, the effect of acute infection and inflammation on functional HSCs is still largely unknown. Here we found that acute infection elicited by heat-inactivated *Escherichia coli* (HIEC) expanded bone marrow lineage-negative (Lin)^−^ stem-cell antigen 1 (Sca-1)^+^cKit^+^ (LSK) cell population, leading to reduced frequency of functional HSCs in LSK population. However, the total number of BM phenotypic HSCs (Flk2^−^CD48^−^CD150^+^ LSK cells) was not altered in HIEC-challenged mice. Additionally, the reconstitution capacity of the total BM between infected and uninfected mice was similar by both the competitive repopulation assay and measurement of functional HSCs by limiting dilution. Thus, occasionally occurring acute inflammation, which is critical for host defenses, is unlikely to affect HSC self-renewal and maintenance of long-term reconstitution capacity. During acute bacterial infection and inflammation, the hematopoietic system can replenish hematopoietic cells consumed in the innate inflammatory response by accelerating hematopoietic stem and progenitor cell proliferation, but preserving functional HSCs in the BM.

## Highlights

- Heat-inactivated *E. coli*-elicited acute infection triggers expansion of lineage^−^sca-1^+^c-kit^+^ (LSK) cells.
- HIEC-elicited acute infection reduces the frequency of functional HSCs in expanded LSK cells but does not alter the total number of functional HSCs in the BM.
- HIEC-induced acute infection does not affect the long-term reconstitution activity of BM HSCs in primary recipients.
- HIEC-induced acute infection does not affect the long-term reconstitution activity of BM HSCs after the secondary BM transplantation.

## Introduction

Blood cell homeostasis is maintained by balanced self-renewal and differentiation of bone marrow (BM) hematopoietic stem and progenitor cells (HSPCs) (1). During steady-state hematopoiesis, most hematopoietic stem cells (HSCs) are quiescent. Hematological stresses such as blood loss, exposure to cytotoxic agents, infection, and inflammation can enhance HSPC proliferation, mobilization, and myeloid differentiation to replenish hematopoietic cells (2–5). These effects are mediated by various pro-inflammatory cytokines such as interferons (INF), tumor necrosis factor (TNFα), interleukin-1 (IL-1), transforming growth factor (TGF)-β, granulocyte-colony stimulating factor (G-CSF), and IL-6 (6–17). Additionally, functional toll-like receptors (TLRs) exist on HSPCs, so bacterial components such as lipopolysaccharide (LPS) may directly stimulate HSPC proliferation (18–22), and HSPCs are often committed to myeloid differentiation after stimulation with TLR ligands (19, 21–23).

Proliferating HSCs often display reduced long-term engraftment and capacity for multilineage reconstitution compared to G0 cells. Thus, it is commonly believed that frequently dividing HSCs are likely functionally impaired (24). Consistently, pathogen-initiated chronic inflammation or autoimmune diseases accelerate proliferation and promote differentiation of HSCs but simultaneously reduce reconstitution capacity (2, 3, 5, 6, 12, 13, 25–29). Repetitive and relatively high doses of cytokines or LPS produce similar outcomes (11, 30–32).

HSCs are thought to divide more frequently in response to infection and inflammation, reducing their reconstitution capability. This conclusion is mainly based on results of pathogen-initiated chronic inflammation or autoimmune diseases (2, 3, 5, 6, 12, 13, 20, 25–29). The effect of acute infection and inflammation on functional HSCs remains elusive. Acute inflammation is an essential host defense against invading viral, bacterial, and fungal infections and a response to tissue injury. A significant number of blood cells, particularly innate immune cells such as monocytes and neutrophils, are consumed in acute inflammation. Accelerated hematopoiesis and myelopoiesis are critical host responses that restore blood homeostasis after acute inflammation. Acute infection and inflammation are frequent events throughout life, but whether this generally beneficial host defense response causes long-term damage to hematopoietic cells is still an open question.

Here we induced acute infection and inflammation using heat-inactivated *Escherichia coli* (HIEC) and determined whether such treatment was detrimental to HSCs. Challenge with HIEC expanded the BM lineage-negative (Lin)^−^ stem cell-antigen 1 (Sca-1)^+^cKit^+^ (LSK) population, which was largely due to upregulation of Sca-1 on LK cells. The total number of BM phenotypic HSCs (Flk2-CD48^−^CD150^+^ LSK cells) was not altered in HIEC-challenged mice. Consistently, there was no significant reduction in reconstitution capacity of the total BM in the infected mice measured by both the competitive repopulation assay and measurement of functional HSCs by limiting dilution. We conclude that occasionally occurring acute inflammation, which is critical for host defenses, is unlikely to affect HSC self-renewal and maintenance of long-term reconstitution capacity.

## Materials and Methods

### Mice

C57BL/6 and C57BL/6/Ly5.1 mice were purchased from the Jackson Laboratory (Bar Harbor, ME). Although sex-based immunological differences are well-documented (33), infection-induced alteration of hematopoietic system and emergency hematopoiesis occur in both males and females. To eliminate age and sex-related variation, we used aged matched (8–12 weeks old) male mice in current study. All mice were housed and cared for in approved veterinary facilities located within the Children's Hospital Boston, which provides sterile isolator cages with fresh food, water, and bedding provided weekly. All animal manipulations were conducted in accordance with the Animal Welfare Guidelines of the Children's Hospital Boston. The Children's Hospital Animal Care and Use Committee approved and monitored all procedures.

### Heat-Inactivated *E. coli*-Elicited Peritoneal Inflammation

To assess the effect of acute infection on HSCs, we developed an acute bacterial infection-induced inflammation model using the Gram-negative bacterium *E. coli*, a pathogen commonly isolated from bacteremic patients. To eliminate the variation caused by differential bacterial growth *in vivo*, we used heat-inactivated *E. coli* (strain 19138, ATCC) (HIEC). HIEC were prepared as previously (34). Briefly, bacteria were first cultured in LB broth at 37°C for 16 h and then washed and re-suspended in PBS. *E. coli* were killed by heating suspensions to 60°C for 1 h. To induce peritoneal inflammation, HIEC (1 × 10^7^ in 200 μl PBS) was injected intraperitoneally. At different time points after HIEC injection, mice were anesthetized with isoflurane and retro-orbital blood was collected. At the end of the experiments, mice were euthanized by CO_2_ inhalation. Inflammation-induced granulopoiesis was assessed by analyzing PB and BM cells.

### Hematologic Analysis

Mice were anesthetized and immediately bled retro-orbitally into an EDTA-coated tube (Becton Dickinson, Franklin Lakes, NJ; Cat: 365974). Complete blood counts were performed using an automated hematology analyzer (Hemavet 850; Drew Scientific, Oxford, CT). For BM cells, the total cell counts were determined using a hemocytometer, and the differential cell counts were conducted by microscopic analysis or FACS analysis using a FACSCanto II flow cytometer (BD Biosciences, San Jose, CA). The absolute numbers of neutrophils and other immune cells were determined based on FACS analysis.

### FACS Analysis

Mice were 8 to 12-week-old males. Single-cell BM suspensions were obtained by re-flushing both tibias and femurs using a 25 G needle and filtering through 40 μm cell strainers. Erythrocytes were lysed with an ACK lysis buffer (Gibco BRL). Single-cell BM and PB cell suspensions were washed with DPBS (Life Technologies, Carlsbad, CA; Cat: 14190-250) supplemented with 2% FCS (Atlanta Biologicals, Flowery Branch, GA; Cat: S11150H). The following antibodies were used for flow cytometry: allophycocyanin-conjugated lineage markers specific for CD3e (145-2C110), CD4 (RM4-5), CD8a (53-6.7), CD11b (M1/70), B220 (RA3-6B2), GR-1 (RB6-8C5), and Ter119 (TER119) (eBioscience, Thermo Fisher Scientific; BioLegend, or BD Pharmingen). Other antibodies included PC-Cy7- or FITC-conjugated Sca-1 (D7), APC-conjugated c-kit (2B8), APC-conjugated CD45.2 (104), PE- conjugated CD150 (SLAM) (clone TC15-12F12.2), FITC-conjugated CD48 (clone HM48-1), and PE-conjugated CD45.1 (A20). Samples were incubated in DMEM (Life Technologies; Cat: 31053-028) supplemented with 2% FCS on ice for 15 min, washed, and filtered before analysis. Unstained cells were used as negative controls to establish the flow cytometer voltage settings, and single-color staining controls were used to adjust the compensation. Unstained cells were used as negative controls to establish the flow cytometer voltage settings, and single-color staining controls were used to adjust the compensation. Flow cytometry was performed on the CANTO II, LSR II, and LSRFortessa (BD Biosciences) instruments. Flow cytometry data were analyzed with FlowJo software (TreeStar).

### Hematopoietic Stem and Progenitor Cell Sorting

Single-cell BM suspensions were obtained by flushing tibias and femurs using a 25 G needle and filtering through 40 μm cell strainers. Erythrocytes were lysed with an ACK lysis buffer (Gibco BRL). Single-cell BM suspensions were washed twice with DPBS (Life Technologies, Carlsbad, CA) supplemented with 2% FCS (Atlanta Biologicals, Flowery Branch, GA). The cell suspension was centrifuged for 5 min at 1,500 rpm, and the cell pellet was resuspended in 1 ml of DPBS with 2% FCS. For sorting, cells were stained with the CD3e (145-2C110), CD4 (RM4-5), CD8a (53-6.7), CD11b (M1/70), B220 (RA3-6B2), GR-1 (RB6-8C5), and Ter119 (TER119) antibodies (eBioscience, Thermo Fisher Scientific, BioLegend, or BD Pharmingen). Other antibodies included PC-Cy7- or FITC-conjugated Sca-1 (D7) and APC-conjugated c-kit (2B8) (BD Pharmingen), LSK cells were sorted using a FACSAria III equipped with FACSDiva software (BD Biosciences).

### BM Cell Transplantation

Donor LSK cells were sorted as described above. For LSK cell transplantation, 8- to 12-week-old CD45. Two recipient mice were lethally irradiated (10.5 Gy, split dose 3–4 h apart) using a ^137^Cs source. The sorted CD45.1+ LSK cells were retro-orbitally injected together with 0.5 million CD45.2 supporting cells into lethally irradiated recipients. For competitive transplantation experiments, 8- to 12-week-old CD45.1 and 2 recipient mice were lethally irradiated (10.5 Gy, split dose 3-4 h apart) using a ^137^Cs source. Half a million CD45.2^+^ donor cells combined with 0.5 million CD45.1^+^ donor cells were injected retro-orbitally. For serial transplantation, secondary recipients were transplanted with one million total BM cells from primary recipients 24 weeks after initial transplantation. The donor whole BM (WBM) cells were prepared by spinning femurs and tibias under sterile conditions. The red blood cells were lysed using ACK lysing buffer. For limiting dilutions, different doses of LSK cells sorted from PBS or HIEC-treated mice were injected together with 0.5 million supporting cells into lethally irradiated recipients. Transplanted mice were first bled 2 or 4 weeks after transplantation and then on a monthly schedule. Peripheral blood was obtained retro-orbitally. Complete blood cells counts were analyzed with the Hemavet hematology system (Drew Scientific, Inc., Miami Lakes, FL). Hematopoietic chimerism was analyzed by fluorescence-activated cell sorting (FACS). Red blood cells were lysed in 2 ml ACK (Gibco, Thermo Fisher Scientific, Waltham, MA) before flow cytometry.

### Analysis of Cell Proliferation by Ki67

Cell proliferation *in vivo* after HIEC-induced inflammation was determined by staining with Ki67-FITC (BD Biosciences, 556026) antibody and Hoechest33342 dye which measure the frequency of cycling cells. BM cells were fixed using the eBioscience Intracellular Fixation and Permeabilization buffer set (Invitrogen). After fixation and permeabilization, cells were centrifuged and resuspended in 100 μl permeabilization buffer. Cells were subsequently stained and analyzed following a protocol provided by the manufacturer (Invitrogen).

### Granulocyte/Monocyte CFU Assays

2 × 10^4^ BM cells from WT mice were seeded in semisolid MethoCult GF M3434 medium (STEMCELL Technologies, Cambridge, UK) containing recombinant mouse stem cell factor (SCF), recombinant mouse IL-3, and recombinant human IL-6 for granulocyte/monocyte CFU (CFU-GM) detection. The number of colonies containing >50 cells was counted on day 7.

### Cytokine Analyses

Mice were euthanized at each indicated time points. The peripheral blood was collected via cardiac puncture and allowed to clot at room temperature for 30 min. Samples were subsequently spun down at 12,000 × g for 10 min to remove blood cells. Supernatants were further centrifuged and filtered using a 0.22 μm filter. The resulting sera were stored at −20°C until use. For cytokine measurement, 75 μl of 2 × diluted sample was analyzed with the Mouse 32-plex Assay, Eve Technology (Calgary, Canada).

### Statistical Analysis

Our research design incorporates rigorous methodological approaches, independent blinded measurements, unbiased data analysis plan, and statistical procedures to minimize technical variability and ensure scientific rigor and reproducibility. Experiments were performed independently at least three times and the data were pooled and analyzed together. For most experiments, comparisons were made using a 2-tailed, unpaired, Student's *t*-test. Data were presented as means (±SD). A *P* ≤ 0.05 was considered statistically significant. Statistical analyses were performed with appropriate software (e.g., GraphPad Prism or SPSS Statistics). No samples or animals subjected to successful procedures and/or treatments were excluded from the analysis. The limdil function in ELDA software was used in the analysis of stem cell frequencies [http://bioinf.wehi.edu.au/software/elda/(35)].

## Results

### HIEC-Induced Acute Inflammation Expands LSK Cell Population

**Figure 1:**
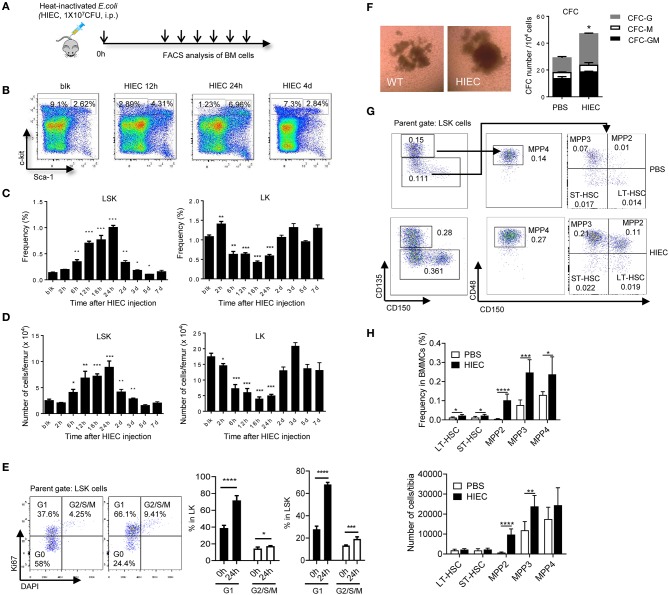
HIEC-elicited acute inflammation triggers expansion of lineage–sca-1+c-kit+ (LSK) cells. **(A)** Experimental scheme for the *in vivo* HI *E. coli* challenge experiments. Heat-inactivated *E. coli* (HIEC, 1 × 10^7^ CFU) were injected intraperitoneally. At each time point, BM cells were collected and analyzed by FACS. **(B)** Representative FACS plots showing changes in LK and LSK cell frequency in BM mononuclear cells at different time points after HIEC challenge. **(C)** Percentage of LSK and LK cells in BM mononuclear cell (BMMC) populations at different time points after *E. coli* challenge. Data shown are means ± SD (*n* = 4 mice). *p < 0.05, **p < 0.01, ***p < 0.001, ****p < 0.0001 vs. unchallenged mice (blk). **(D)** Absolute number of LSK and LK cells in the total BM at different time points after *E. coli* challenge. Data shown are means ± SD (*n* = 4 mice). *p < 0.05, **p < 0.01, ***p < 0.001, ****p < 0.0001 vs. unchallenged mice (blk). **(E)** Percentage of G1 and G2/S/M cells in LK and LSK cells 24 h after the HI *E. coli* challenge. Data shown are means ± SD (*n* = 5 mice). *p < 0.05, **p < 0.01, ***p < 0.001 vs. unchallenged mice (time 0). **(F)** The number of committed myeloid progenitors analyzed using a quantitative granulocyte–monocyte colony-forming unit (CFU-GM) assay. BMMCs were prepared 24 h after *E. coli* injection and cultured in MethoCult™ GF M3434 medium for 7 days. Representative photographs of cell clusters/colonies are shown. The number of granulocyte colony-forming units (CFU-G), monocyte colony-forming units (CFU-M), and granulocyte–monocyte colony-forming units (CFU-GM) from 10,000 BMMCs were calculated. Data shown are mean ± SD of *n* = 3 mice. *p < 0.01 vs. PBS-treated mice. **(G)** Representative FACS plots showing HSC/HPC subpopulations. LT-HSC (CD135^−^CD150^+^CD48^−^LSK), ST-HSC (CD135^−^CD150^−^CD48^−^LSK), MPP2 (CD135^−^CD150^+^CD48^+^LSK, myeloid-biased), MPP3 (CD135^−^CD150^−^CD48^+^LSK, myeloid-biased), and MPP4 (CD135^+^CD150^−^CD48^+^LSK, lymphoid-primed) cell populations were defined as previously reported (54). **(H)** Percentage of LT-HSC, ST-HSC, MPP2, MPP3, and MPP4 cells in BM mononuclear cell (BMMC) population and their absolute number in tibia 24 h after the HI *E. coli* challenge. Data shown are means ± SD (*n* = 5–7 mice). *p < 0.05, **p < 0.01, ***p < 0.001, ****p < 0.0001 vs. control (PBS treated).

To assess the effect of acute infection and inflammation on HSCs, we developed a mouse acute bacterial infection model using heat-inactivated *E. coli* (HIEC) [(36); Figure 1A and Figure S1A]. Peritoneal infection with HIEC triggered mobilization of neutrophils from the BM to the peripheral blood, leading to initial increase of peripheral blood neutrophil counts days (Figure S1B). The number of BM mononuclear cells (BMMCs) reduced at the early time points and then increased after 2 days (Figure S1C). Consistently, peripheral blood counts, particularly neutrophil counts, was elevated after 2 days (Figure S1B). HIEC-challenge also increased spleen size (Figure S1D). In parallel, there was an increase in cytokine production in HIEC-challenged mice (Figure S1E). Consistent with published results, HIEC-elicited acute infection also expanded the HSC-enriched LSK pool (Figure 1B). The percentage (Figure 1C) and total number (Figure 1D) of BM LSK cells increased gradually, peaking 24 h after *E. coli* challenge and returning to baseline after 5 days. Previous study revealed that HIEC-challenge promotes cell division of LSK cells (34). Here, we assessed cell proliferation by staining cells with Ki67 and Hoechest33342. Consistently, the percentage of G1- and G2/S/M-phase proliferating cells increased both in LSK and Lin-Sca-1-c-Kit+ (LK) cell populations (Figure 1E). Bacterial infection triggers myelopoiesis (34, 37–39) and consistent with this, the BM from HIEC-treated mice contained more committed myeloid progenitors than untreated controls (Figure 1F). Thus, HIEC-induced acute infection significantly expands the LSK cell population and augments myelopoiesis.

### HIEC-Induced Acute Inflammation Does Not Alter the Total Number of Functional HSCs in the BM

Mouse HSCs are often characterized as lineage-negative LSK cells, but < 10% of these cells are functional long-term HSCs (LT-HSCs) (40, 41). LSK population also contains short-term HSC with multilineage potential (ST-HSC and MPP). Expansion of LSK cells does not equivalent to expansion of functional HSC population. Additionally, although expansion of the BM LSK population is a hallmark of infections induced by various pathogens including bacteria, fungi, and viruses (5, 9, 42–47), it is well-documented that Sca-1, a canonical stem cell marker, can be upregulated on hematopoietic cells including non-HSCs during inflammation by either IFNs or TNFα (10, 48–50). LPS and IL6 also convert LK to LSK cells *in vitro* (50). Thus, infection-induced expansion of the LSK population may simply reflect elevated Sca-1 expression on LK cells rather than expansion of Sca-1-positive functional HSCs. Significantly higher stem cell purities can be achieved by selecting LSK cells that show differential expression of CD135 (a.k.a. FLT3 and FLK2) and SLAM receptors (CD150 and CD48) (51–53). To better understand the effect of HI *E. coli* on phenotypic HSCs, we furtherly examined the LSK subpopulation, including LT-HSC (CD135^−^CD150^+^CD48^−^LSK), ST-HSC (CD135^−^^−^CD48^−^LSK), MPP2 (CD135^−^CD150^+^CD48^+^LSK, myeloid-biased), MPP3 (CD135^−^CD150^−^CD48^+^LSK, myeloid-biased), and MPP4 (CD135^+^CD150^−^CD48^+^LSK, lymphoid-primed) cells [(54); Figure 1G]. We found that HIEC-triggered expansion of LSK cells mainly occurred in MPP2 and MPP3 populations. The total number of BM LT-HSCs (CD135^−^CD150^+^CD48^−^LSK) that contain long-term, multi-lineage repopulating cells was not increased during HIEC-induced acute inflammation (Figure 1H).

**Figure 2:**
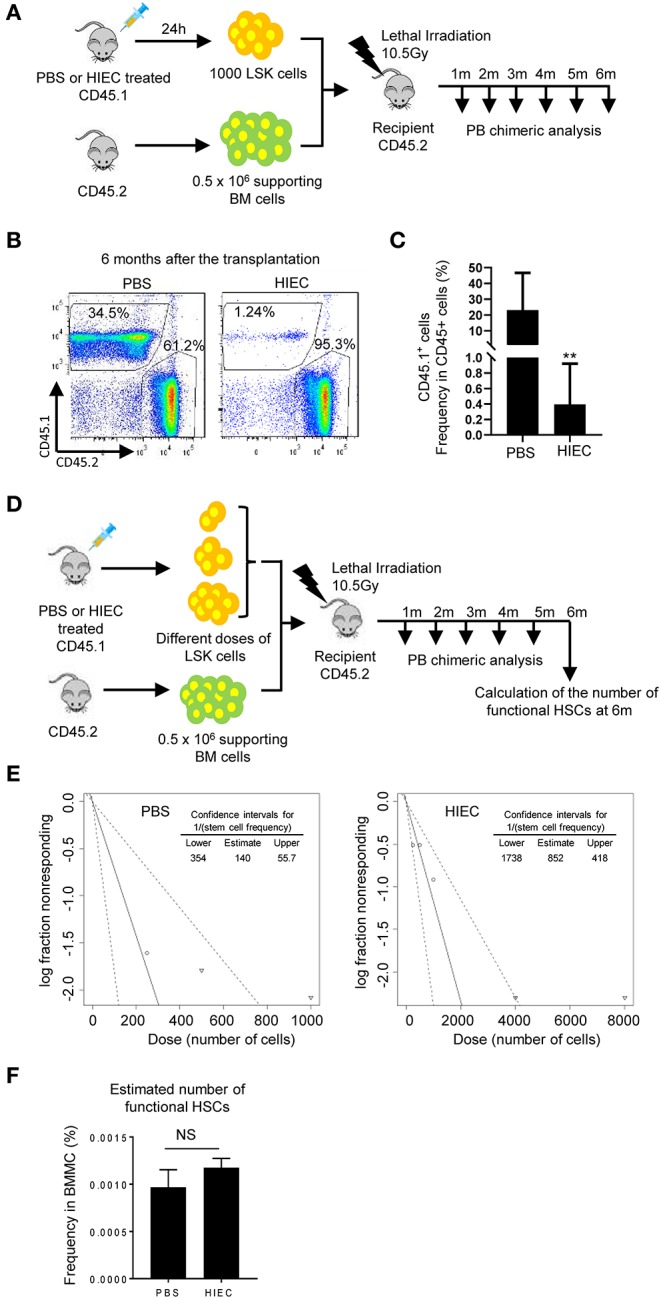
HIEC-elicited acute inflammation reduces the frequency of functional HSCs in expanded LSK cells but does not alter the total number of functional HSCs in the BM. **(A)** Schematic of the *in vivo* hematopoiesis reconstitution experiment. 1000 LSK cells from PBS or HIEC-challenged mice (CD45.1) were transplanted into lethally-irradiated congenic recipients (CD45.2) together with 0.5 million supporting cells (CD45.2). BM chimerism in recipients was analyzed at each indicated time point. **(B)** Representative FACS plots of donor-derived WBCs in the peripheral blood 6 months after transplantation. **(C)** The percentages of donor derived cells (CD45.1^+^) in total hematopoietic cells (CD45.2^+^ or CD45.1^+^) at the indicated time points in PBS and HIEC-treated groups. Data shown are means ± SD (*n* = 5 mice). **p < 0.01 vs. control by unpaired two-tailed nonparametric *t*-test. **(D)** Schematic of the limiting dilution analysis (LDA). Limiting dilution experiments were performed with indicated doses of LSK cells obtained from PBS- or HIEC-treated mice (CD45.1) combined with 5 × 10^5^ competing cells (CD45.2) transplanted into recipients (CD45.2). BM chimerism in recipients was analyzed at each indicated time point. Hematopoietic chimerism was analyzed by FACS. **(E)** Log-fraction plots of the limiting dilution model fitted to the data in Figure S2B and Table S1. Recipient chimerism was determined at 6 months, and >0.5% engraftment was considered positive. The slope of the line is the log-positive transplantation fraction. The dotted lines give the 95% confidence intervals. The frequencies of functional HSCs in LSK cells were calculated accordingly. All the analyses and calculations were performed using ELDA software. **(F)** The number of functional HSCs in the BM was assessed using limiting dilution analysis. The number of functional HSCs was calculated based on the raw data shown in Table S1. Data shown are mean ± SD of *n* = 4 mice. ^NS^*p* > 0.05 vs. PBS-treated mice.

To further examine the function of the expanded BM LSK cells, a competitive BM transplant experiment was performed using LSK cells isolated from HIEC-infected or uninfected CD45.1 mice (Figure 2A). One thousand CD45.1 LSK cells were mixed with 0.5 million total CD45.2 BM cells isolated from uninfected mice and transplanted into lethally-irradiated primary recipients. Donor LSK cells from both uninfected and infected mice engrafted the BM. However, when donor LSK cells were isolated from infected mice, their engraftment efficiency was significantly reduced, remaining at low levels 6 months after transplantation (Figures 2B,C). To further examine whether the number of functional HSCs was reduced in the LSK population isolated from infected mouse BM, we measured competitive repopulating units (CRU) using a limiting dilution assay (Figure 2D). Lethally-irradiated recipient mice were transplanted with different doses of donor LSK cells isolated from infected or uninfected CD45.1 mice together with a constant number (5 × 10^5^) of CD45.2 supporting BM cells. BM chimerism was calculated as the percentage of donor-derived cells in the peripheral white blood cell pool 6 months after transplantation, with chimerism of >0.5% considered positive engraftment (Figure S2 and Table S1). Compared to LSK cells isolated from uninfected donors, CRU frequency in LSK cells isolated from infected donors was significantly decreased from one in 140 to one in 852 (Figure 2E and Table S2). However, since the percentage of LSK cells in the HIEC-challenged mice was seven-fold greater than in unchallenged mice, the total number of CRU per femur was in fact not altered in challenged mice (Figure 2F and Table S2). Taken together, our results show that the frequency of functional HSCs in the expanded LSK cell population appears to be reduced in HIEC-infected mice but the total number is unaltered.

### HIEC-Induced Acute Inflammation Does Not Impair HSC Long-Term Reconstitution Activity in Primary Recipients

**Figure 3:**
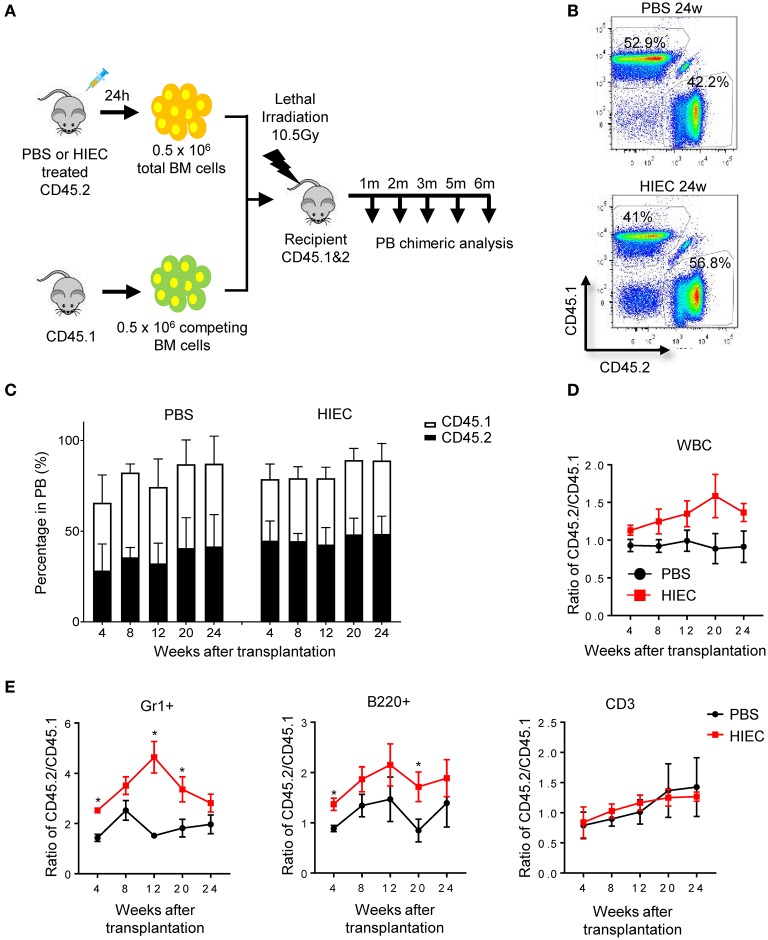
HIEC-induced acute inflammation does not affect the long-term reconstitution activity of BM HSCs in primary recipients. **(A)** Schematic of the competitive BM transplantation experiment. 0.5 million total BM cells from PBS-treated or HIEC-challenged mice (CD45.2) were transplanted into lethally-irradiated congenic recipients (CD45.1 and 2 C57/B6 mice) together with 0.5 million competing BM cells (CD45.1). BM chimerism in recipients was analyzed at each indicated time point. Hematopoietic chimerism was analyzed by FACS. **(B)** Representative FACS plots showing the percentage of donor-derived WBCs in the peripheral blood 24 weeks after transplantation in PBS and HIEC groups. The experiments were conducted 24 weeks after the transplantation. **(C)** The percentage of the donor-derived CD45.2^+^ and CD45.1^+^ WBCs in the peripheral blood at the indicated time points. Data shown are mean ± SD of *n* = 5 mice. **(D)** Ratio of CD45.2^+^ to CD45.1^+^ cells at the indicated time points after transplantation in PBS and HIEC groups. Data shown are mean ± SD of *n* = 5 mice. **(E)** Chimerism in indicated blood lineages. Ratio of CD45.2^+^ to CD45.1^+^ cells was calculated based on the percentage of donor-derived CD45.2^+^ and CD45.1^+^ cells in each indicated linage (Figure S3). Data shown are mean ± SD of *n* = 5 mice. *p < 0.05 vs. PBS-treated group.

Next, to directly measure the total BM reconstitution activity in infected and uninfected mice, we conducted a competitive BM transplant experiment using total BM instead of isolated LSK cells (Figure 3A). BM cells from infected and uninfected mice were mixed at a 1:1 ratio and transplanted into lethally-irradiated primary recipients. After 24 weeks, the majority of recipient peripheral blood cells were CD45.1^+^ or CD45.2^+^, indicating successful engraftment of donor BM cells (Figure 3B). At 4 weeks, the percentage of CD45.2^+^ donor-derived cells (from infected donor mice) was significantly higher than that of CD45.1^+^ donor-derived cells (from uninfected donor mice), indicating BM hematopoietic progenitor cell expansion in the infected donors and that acute infection could indeed induce proliferation of hematopoietic progenitor cells (Figures 3C,D). HSPCs differentiated into a diverse range of specialized cell types, with myeloid lineage cells predominating, consistent with augmented myelopoiesis during acute inflammation [(3, 55, 56); Figure 3E and Figure S3] and the elevated G/M-CFU detected in infected mice. To evaluate the long-term engraftment of transplanted HSCs, peripheral blood was continuously sampled at 4-week intervals for 24 weeks, over which time the percentage of CD45.2^+^ cells did not fall (Figures 3C,D). In fact, a higher, although sometimes not statistically significant, percentage of CD45.2^+^ cells compared to CD45.1^+^ cells was always detected. The unaltered or even elevated CD45.2^+^ cell percentage represented all cell types including B cells (B220^+^), T cells (CD3^+^), and myeloid cells (Gr1^+^). Therefore, the number and function of long-term multiple-lineage BM HSCs was likely unaltered or even elevated in acute inflammation (Figure 3E and Figure S3).

### HIEC-Induced Acute Inflammation Does Not Impair HSC Long-Term Reconstitution Activity After the Secondary BM Transplantation

**Figure 4:**
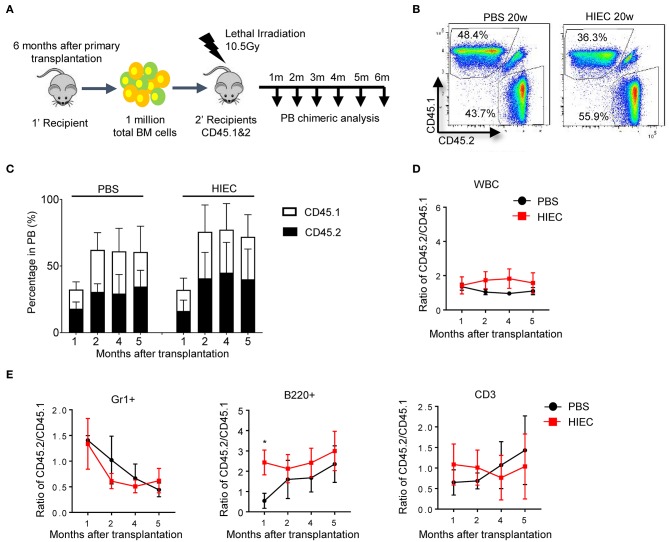
HIEC-induced acute inflammation does not affect the long-term reconstitution activity of BM HSCs after the secondary BM transplantation. **(A)** Schematic of secondary BM transplantation experiment. One million total BM cells from primary recipients were transplanted into lethally-irradiated congenic recipients (CD45.1 and 2 C57/B6 mice). BM chimerism in secondary recipients was analyzed at each indicated time point. Peripheral blood was obtained retro-orbitally using a heparinized capillary tube. Hematopoietic chimerism was analyzed by FACS. **(B)** Representative FACS plots showing the percentage of CD45.2^+^ and CD45.1^+^ WBCs in the peripheral blood 20 weeks after secondary BM transplantation. The experiments were conducted 20 weeks after the transplantation. **(C)** The percentage of donor-derived CD45.2^+^ and CD45.1^+^ WBCs in the PB of secondary recipients at the indicated time points. Data shown are mean ± SD of *n* = 5 mice. **(D)** Ratio of CD45.2^+^ to CD45.1^+^ cells at the indicated time points after secondary BM transplantation. Data shown are mean ± SD of *n* = 5 mice. **(E)** Chimerism in indicated blood lineages. Ratio of CD45.2^+^ to CD45.1^+^ cells was calculated based on the percentage of donor-derived CD45.2^+^ and CD45.1^+^ cells in each indicated linage (Figure S4). Data shown are mean ± SD of *n* = 5 mice. *p < 0.05 vs. PBS-treated group.

To further reveal how acute inflammation affects HSC function in the long term, we conducted secondary transplantation of BM cells from primary recipients (Figure 4A). Total BM cells were harvested from primary recipients after 4 months and then transplanted into lethally-irradiated secondary recipients. In contrast to primary recipients, the peripheral blood of secondary recipients did not show further increases in CD45.2^+^-derived cells. The ratio of CD45.2^+^ to CD45.1^+^ cells remained constant over the 6-month period (Figures 4B–D). At each time-point examined over long-term serial transplantation, secondary recipients showed long-term multilineage reconstitution derived from both CD45.2^+^ and CD45.1^+^ donor cells. Compared to the donor population ratio, there was no significant reduction in CD45.2:CD45.1 cell ratio for each lineage (Figure 4E and Figure S4). Noticeably, HIEC challenge was able to significantly increase the reconstitution capacity of myeloid cells and B cells at several time points post the first transplantation (Figure 3E). Similar effect was also observed in B cell reconstitution 1 month after the secondary BM transplantation (Figure 4E). This was likely due to the expansion of hematopoietic progenitor cells and/or ST-HSCs which were enriched in the LSK population [(34); Figure 1]. However, such effect became significant 2 month after the secondary BM transplantation (Figure 4E). Collectively, our results demonstrate that HIEC-induced acute inflammation expands the BM LSK population but does not affect the long-term reconstitution activity of BM HSCs.

## Discussion

### HIEC-Induced Acute Inflammation Model

To assess the effect of acute infection and inflammation on HSCs, we developed a mouse acute bacterial infection model using the Gram-negative bacterium *E. coli* K12, a strain commonly isolated from bacteremic patients. *E. coli* normally resides in the intestine but is a major cause of sepsis in hospitalized patients (36). Live bacteria grow in the host, introducing an unnecessary variable to the system. To eliminate the variation caused by differential bacterial growth *in vivo*, we used heat-inactivated bacteria (36). Additionally, the focus of current study is the acute response to infection and inflammation. A controlled and consistent acute infection is better achieved by using heat-inactivated *E. coli*. If mice were challenged with live *E. coli*, the infection would last much longer. The function of HSPCs isolated from mice infected with live *E. coli* for 6 days has been previously examined, and the total BM HSC activity in *E. coli-*infected mice was moderately diminished compared to that of uninfected mice, mainly due to HSC mobilization from the BM to the spleen (57).

### The Effect of Acute Infection on HSC Function

In this study, we investigated the impact of acute infection on HSC function using the HIEC-induced acute infection model. Challenge with HIEC expanded the BM LSK cell population, which was largely due to upregulation of Sca-1 on LK cells. The total number of BM phenotypic HSCs (Flk2^−^/CD48^−^/CD150^+^ LSK cells) was not altered in HIEC-challenged mice. Consistently, there was no significant reduction in reconstitution capacity of the total BM in the infected mice assessed by both the competitive repopulation assay and measurement of functional HSCs by limiting dilution. Noticeably, a similar result was observed in terms of the role of type I interferons in hematopoiesis (10). When CD150^+^ LSK was used as phenotypic definition for HSCs, interferon signaling led to expansion of HSCs (8, 58). In contrast, when the more stringent Flk2^−^/CD48^−^/CD150^+^ LSK was used as phenotypic definition for HSCs, IFN–induced HSC proliferation was only a transient event even in the presence of chronic IFN treatment, and was not sufficient to deplete the HSC pool (10).

Few studies have assessed the impact of acute infection on HSC function. In a *Pseudomonas aeruginosa-*induced sepsis model, LSK compartment expansion was observed in the first 24 h of sepsis, and there was reduced engraftment of LSK cells from infected animals after transplantation into lethally-irradiated recipient mice. It was therefore concluded that acute bacterial infection reduces stem cell activity and reconstitution potential (44). Nevertheless, in this study, the competitive BM transplantation experiments were conducted using the same number of LSK cells from infected and uninfected control mice. The reduced BM reconstitution capacity of LSK cells from infected mice might simply be caused by increased Sca-1 expression in progenitor cells and thus LT-HSC dilution by progenitor cells. Further, in a bacterial infection by *Staphylococcus aureus* model and a cecal ligation and puncture (CLP) acute polymicrobial sepsis model (46), the LSK population was expanded. The number of CD150^+^CD135^−^ LSK cells which include LT-HSC (CD135^−^CD150^+^CD48^−^LSK) and MPP2 (CD135^−^CD150^+^CD48^+^LSK) also increased, consistent with the significant expansion of MMP2 (Figure 1H). CD150^+^CD135^−^ cells from septic mice retained sufficient reconstitution capacity to allow survival of lethally-irradiated recipients; however, engraftment efficiencies were not compared between HSCs from infected and uninfected mice. Interestingly, CLP-induced HSPC expansion still occurred in the absence of TLR signaling (46).

It is noteworthy that acute viral infection also induces transient alterations on the hematopoietic process (59, 60). Direct viral infection in HSPCs has been shown to reduce the hematopoietic output. However, the exact underlying mechanisms is largely unknown. Additionally, viral infection can affect hematopoiesis through the action of mediators such as type I IFNs, TNFα, and other cytokines generated during the infection (59, 60). It was previously reported that vaccinia virus infection induces MyD88-dependent expansion of the both Flk-2^+^ and Flk-2^−^ LSK cell populations in the BM (5). Its effect on the long-term BM reconstitution capability of functional HSCs remains elusive.

### Mechanisms That Maintains the Reconstitution Capability of HSCs During HIEC-Induced Acute Inflammation

Bacterial components such as Lipopolysaccharide (LPS) and inflammatory cytokines can stimulate HSPC proliferation (6–22). Both Flk-2^−^ (long-term repopulating HSC enriched) and Flk-2^+^ LSK cells express TLR4 (21, 61). LPS interacts directly with the TLR4 on these cells in bone marrow, triggering cell cycle entry and stimulating innate immune system replenishment in challenged mice (21). Chronic low-dose LPS stimulation leads to HSPC expansion and common lymphoid progenitor (CLP) depletion. HSC and CLP sensitivity to LPS depends on hematopoietic-derived, cell subset–autonomous TLR4 (20). Takizawa et al. also revealed that systemic LPS application enhances HSC (LKS CD34^−^CD150^+^) proliferation directly via TLR4 (31). TLR1/2 agonist PAM3CSK4 also promotes proliferation of both human HSCs (Lin^−^CD34^+^CD38^lo^) and HPCs (Lin^−^CD34^+^CD38^hi^). It can bias the lineage commitment of human HSCs and shift the differentiation of lineage-committed progenitors to favor myelopoiesis at the expense of lymphoid B-cell development (19). Similarly, specific ligands for TLR7/8 induce the proliferation and differentiation of human BM CD34^+^ progenitor cells along the myeloid Lineage (22). Noticeably, HSPCs can produce various cytokines in response to LPS and Pam3CSK4 stimulation. Among these cytokines, IL-6 is a particularly important regulator of myeloid differentiation and HSPC proliferation and in mediating rapid myeloid cell recovery during neutropenia (61). Interferon signaling also plays critical roles in hematopoiesis. In response to interferon-a (IFNa) or poly(I:C), an inducer of type I IFN signaling, HSCs [CD150^+^LSK cells (8, 58) or CD150^+^CD41^−^CD48^−^ LSK cells (14)] efficiently exit G0 and enter an active cell cycle, leading to expansion of HSC population. Interferon γ (IFNγ) promotes proliferation of LSK cell both *in vivo* and *in vitro* (10, 17). In an *in vivo* mouse model of *Mycobacterium avium* infection, chronic infection triggers IFNγ-mediated proliferation of both LT-HSCs (CD34^−^Flk2^−^ LSK or CD150^+^LSK) and ST-HSCs (CD34^+^Flk2^−^LSK) (6). It was later reported that expansion of HSPCs (LSK cells) triggered by intracellular bacterial infection is mainly mediated by IFNγ. In a murine model of human monocytic ehrlichiosis, IFNγ but not IFN-a, acts directly on hematopoietic cells to promote their proliferation leading to increased frequency of LSK cells in *Ehrlichia muris*-infected hosts (9). Interestingly, another study showed that IFNγ secreted by effector cytotoxic CD8^+^ T cells can promote the release of hematopoietic cytokines, such as IL-6 from bone marrow mesenchymal stromal cells (MSCs), which in turn stimulates hematopoiesis at the level of early multipotent hematopoietic progenitor cells (Lin^−^c-kit^hi^) (15). Finally, interleukin-1 (IL-1) has also been implicated in regulating HSC proliferation. Chronic IL-1 exposure accelerates cell division and myeloid differentiation of HSCs (Flk2^−^CD48^−^CD150^+^ LSK cells) through precocious activation of a PU.1-dependent gene program (11).

It is commonly thought that HSCs divide more frequently in response to infection, leading to reduced reconstitution capability (24), a phenomenon known as proliferation-associated functional exhaustion (62). In an early study, Passegue et al. functionally assessed the proliferation index and status of cell cycle machinery during hematopoietic differentiation and demonstrated that hematopoietic repopulating potential critically depends on maintenance of the quiescent state (24). Similar effect was also observed in a bacterial infection model in which *in vivo* LPS application induces proliferation of dormant HSCs via TLR4 and sustained LPS exposure impairs HSC self-renewal and competitive repopulation activity (31). Chronic type I IFN signaling induces HSC proliferation but also leads to functional exhaustion of quiescent HSCs (8, 14). Similarly, chronic IL-1 exposure reduces the self-renewal capability of HSC (11). Thus, our finding that *E. coli*-induced acute infection and inflammation does not affect the reconstitution capacity in BM is somewhat surprising, suggesting that there must be an intrinsic biological mechanism that maintains the reconstitution capability of HSCs during acute infection.

First, it is possible that the functional LT-HSCs do not divide during acute infection and inflammation. Despite the fact that chronic inflammation augments HSC proliferation and self-renewal (2, 3, 5, 6, 12, 13, 25–29), there is no definitive evidence showing that acute infection also increases HSC proliferation. BM LSK cells expand during acute infection, which may simply be a result of infection-induced Sca-1 expression on ST-HSC and progenitor cells (LK cells) (48–50). Similarly, the accelerated LSK cell division may simply be a result of accelerated division of ST-HSC and progenitor cells. HSCs express TLRs and various cytokine receptors. LPS and cytokines can induce HSC proliferation both *in vitro* and *in vivo* (6–19, 21, 22). On the other hand, cytokines such as TNFα may act as a cell extrinsic and potent suppressor of normal HSC activity (12, 13). Numerous cytokines and chemokines are produced during acute and chronic infection. *E. coli* can upregulate various proinflammatory cytokine expression in cultured human CD34^+^ cells (63). Some of these cytokines may antagonize the cell division-promoting effect of LPS and cytokines. Acute infection may also indirectly modulate the BM microenvironment, making it unfavorable for HSC proliferation. In this model, HSCs would not proliferate in response to acute infection and inflammation and would thus maintain their long-term reconstitution capability.

Second, the host may develop a strategy to maintain the “stem” capacity of dividing HSCs. Acute infection increases HSC proliferation as in chronic inflammation (2, 3, 5, 6, 12, 13, 25–29), but the transiently proliferating HSCs can maintain their long-term reconstitution capacity. In this situation, increased HSC proliferation and differentiation would not necessarily impair HSC functionality, perhaps due to the action of certain cytokines/chemokines or a unique BM microenvironment generated during acute or chronic infection. In this scenario, the total long-term reconstitution activity would be increased in infected mice. Notably, we only observed a small (not statistically significant) increase in the reconstitution activity in the total BM of infected mice compared to uninfected mice. This might be a result of HSC mobilization from the BM to peripheral blood and spleen during acute infection, thus reducing the number of BM HSCs. It is well-known that infection or systemic administration of LPS mobilizes HSPCs (57, 64–66).

Finally, another non-exclusive possibility is that the size and number of bone marrow niches, which determine the number of BM HSCs, are only impaired during chronic but not acute infection and inflammation. Quiescent HSCs are lodged in phenotypically-defined BM niches, and their self-renewal and differentiation are tightly controlled by this surrounding microenvironment (67–70). Even though acute inflammation triggers proliferation and therefore increases HSC number, the HSCs outside the niches lose their reconstitution activity and only HSCs in the niches retain their reconstitution activity, resulting in unaltered HSC numbers in infected mice.

### The Evolutionary Perspective and Clinical Implications

Uncontrolled inflammation in chronic infection and autoimmune diseases is not physiologically designed, so it is understandable that HSCs deplete and suffer long-term functional defects such as those seen in certain acquired BM failure syndromes. By contrast, acute inflammation in response to pathogens is an essential and evolutionarily selected host defense mechanism that ensures rapid clearance of invading pathogens. Our findings suggest that, during acute infection and inflammation, the hematopoietic system can replenish hematopoietic cells consumed during the innate inflammatory response by accelerating HSPC proliferation. However, this acute process preserves functional HSCs in the BM. Therefore, from the clinical perspective, an induced acute inflammatory response may be used to improve the efficacy of BM recovery after chemo- or radiotherapy without compromising the long-term repopulating capability of HSCs. We focused on *E. coli*-elicited acute infection in this study, but it should be noted that hematopoietic responses to infection are pathogen dependent, relying on the distinct pathogen-associated molecular patterns (PAMPS), cytokines, and chemokines elicited by different pathogens (2, 71). Whether other Gram-negative and Gram-positive bacteria, viruses, or fungi have the same effect on HSCs remains to be determined.

## Conclusion

Taken together, the results of our work demonstrate that occasionally occurring acute inflammation, which is critical for host defenses, is unlikely to affect HSC self-renewal and maintenance of long-term reconstitution capacity. During acute bacterial infection and inflammation, the hematopoietic system can replenish hematopoietic cells consumed in the innate inflammatory response by accelerating hematopoietic stem and progenitor cell proliferation, but preserving functional HSCs in the BM.

## Data Availability Statement

All datasets generated for this study are included in the article/Supplementary Material.

## Ethics Statement

This animal study was reviewed and approved by Children's Hospital Animal Care and Use Committee.

## Author Contributions

XZ, KK, DC, HZ, RG, and HL conceived of the project. XZ, KK, DC, HZ, RG, PL, HY, QR, and XL designed, performed the experiments, and analyzed the data. XZ and HL wrote the manuscript. HL, YX, FM, MH, and TC supervised the project. HL acquired the funds. All authors contributed to the review of the manuscript.

### Conflict of Interest

The authors declare that the research was conducted in the absence of any commercial or financial relationships that could be construed as a potential conflict of interest.

## References

[1] Kondo M Wagers AJ Manz MG Prohaska SS Scherer DC Beilhack GF. Biology of hematopoietic stem cells and progenitors: implications for clinical application. Annu Rev Immunol. (2003) 21:759–806. 10.1146/annurev.immunol.21.120601.141007 12615892

[2] Glatman Zaretsky A Engiles JB Hunter CA. Infection-induced changes in hematopoiesis. J Immunol. (2014) 192:27–33. 10.4049/jimmunol.1302061 24363432 PMC3874119

[3] King KY Goodell MA. Inflammatory modulation of HSCs: viewing the HSC as a foundation for the immune response. Nat Rev Immunol. (2011) 11:685–92. 10.1038/nri3062 21904387 PMC4154310

[4] Shahbazian LM Quinton LJ Bagby GJ Nelson S Wang G Zhang P. *Escherichia coli* pneumonia enhances granulopoiesis and the mobilization of myeloid progenitor cells into the systemic circulation. Crit Care Med. (2004) 32:1740–6. 10.1097/01.CCM.0000132900.84627.90 15286552

[5] Singh P Yao Y Weliver A Broxmeyer HE Hong SC Chang CH. Vaccinia virus infection modulates the hematopoietic cell compartments in the bone marrow. Stem Cells. (2008) 26:1009–16. 10.1634/stemcells.2007-0461 18258722 PMC2814369

[6] Baldridge MT King KY Boles NC Weksberg DC Goodell MA. Quiescent haematopoietic stem cells are activated by IFN-gamma in response to chronic infection. Nature. (2010) 465:793–7. 10.1038/nature09135 20535209 PMC2935898

[7] Challen GA Boles NC Chambers SM Goodell MA. Distinct hematopoietic stem cell subtypes are differentially regulated by TGF-beta1. Cell stem cell. (2010) 6:265–78. 10.1016/j.stem.2010.02.002 20207229 PMC2837284

[8] Essers MA Offner S Blanco-Bose WE Waibler Z Kalinke U Duchosal MA. IFNalpha activates dormant haematopoietic stem cells *in vivo*. Nature. (2009) 458:904–8. 10.1038/nature07815 19212321

[9] MacNamara KC Oduro K Martin O Jones DD McLaughlin M Choi K. Infection-induced myelopoiesis during intracellular bacterial infection is critically dependent upon IFN-gamma signaling. J Immunol. (2011) 186:1032–43. 10.4049/jimmunol.1001893 21149601 PMC3178067

[10] Pietras EM Lakshminarasimhan R Techner JM Fong S Flach J Binnewies M. Re-entry into quiescence protects hematopoietic stem cells from the killing effect of chronic exposure to type I interferons. J Exp Med. (2014) 211:245–62. 10.1084/jem.20131043 24493802 PMC3920566

[11] Pietras EM Mirantes-Barbeito C Fong S Loeffler D Kovtonyuk LV Zhang S. Chronic interleukin-1 exposure drives haematopoietic stem cells towards precocious myeloid differentiation at the expense of self-renewal. Nat Cell Biol. (2016) 18:607–18. 10.1038/ncb3346 27111842 PMC4884136

[12] Pronk CJ Veiby OP Bryder D Jacobsen SE. Tumor necrosis factor restricts hematopoietic stem cell activity in mice: involvement of two distinct receptors. J Exp Med. (2011) 208:1563–70. 10.1084/jem.20110752 21768269 PMC3149225

[13] Rebel VI Hartnett S Hill GR Lazo-Kallanian SB Ferrara JL Sieff CA. Essential role for the p55 tumor necrosis factor receptor in regulating hematopoiesis at a stem cell level. J Exp Med. (1999) 190:1493–504. 10.1084/jem.190.10.1493 10562323 PMC2195701

[14] Sato T Onai N Yoshihara H Arai F Suda T Ohteki T. Interferon regulatory factor-2 protects quiescent hematopoietic stem cells from type I interferon-dependent exhaustion. Nat Med. (2009) 15:696–700. 10.1038/nm.1973 19483695

[15] Schurch CM Riether C Ochsenbein AF. Cytotoxic CD8+ T cells stimulate hematopoietic progenitors by promoting cytokine release from bone marrow mesenchymal stromal cells. Cell Stem Cell. (2014) 14:460–72. 10.1016/j.stem.2014.01.002 24561082

[16] Ueda Y Cain DW Kuraoka M Kondo M Kelsoe G. IL-1R type I-dependent hemopoietic stem cell proliferation is necessary for inflammatory granulopoiesis and reactive neutrophilia. J Immunol. (2009) 182:6477–84. 10.4049/jimmunol.0803961 19414802 PMC2780360

[17] Zhao X Ren G Liang L Ai PZ Zheng B Tischfield JA. Brief report: interferon-gamma induces expansion of Lin(-)Sca-1(+)C-Kit(+) cells. Stem Cells. (2010) 28:122–6. 10.1002/stem.252 19890981

[18] Boettcher S Ziegler P Schmid MA Takizawa H van Rooijen N Kopf M. Cutting edge: LPS-induced emergency myelopoiesis depends on TLR4-expressing nonhematopoietic cells. J Immunol. (2012) 188:5824–8. 10.4049/jimmunol.1103253 22586037

[19] De Luca K Frances-Duvert V Asensio MJ Ihsani R Debien E Taillardet M. The TLR1/2 agonist PAM(3)CSK(4) instructs commitment of human hematopoietic stem cells to a myeloid cell fate. Leukemia. (2009) 23:2063–74. 10.1038/leu.2009.155 19641520

[20] Liu A Wang Y Ding Y Baez I Payne KJ Borghesi L. Cutting edge: hematopoietic stem cell expansion and common lymphoid progenitor depletion require hematopoietic-derived, cell-autonomous TLR4 in a model of chronic endotoxin. J Immunol. (2015) 195:2524–8. 10.4049/jimmunol.1501231 26276875 PMC4561199

[21] Nagai Y Garrett KP Ohta S Bahrun U Kouro T Akira S. Toll-like receptors on hematopoietic progenitor cells stimulate innate immune system replenishment. Immunity. (2006) 24:801–12. 10.1016/j.immuni.2006.04.008 16782035 PMC1626529

[22] Sioud M Floisand Y Forfang L Lund-Johansen F. Signaling through toll-like receptor 7/8 induces the differentiation of human bone marrow CD34+ progenitor cells along the myeloid lineage. J Mol Biol. (2006) 364:945–54. 10.1016/j.jmb.2006.09.054 17049554

[23] Megias J Yanez A Moriano S O'Connor JE Gozalbo D Gil ML. Direct Toll-like receptor-mediated stimulation of hematopoietic stem and progenitor cells occurs *in vivo* and promotes differentiation toward macrophages. Stem Cells. (2012) 30:1486–95. 10.1002/stem.1110 22511319

[24] Passegue E Wagers AJ Giuriato S Anderson WC Weissman IL. Global analysis of proliferation and cell cycle gene expression in the regulation of hematopoietic stem and progenitor cell fates. J Exp Med. (2005) 202:1599–611. 10.1084/jem.20050967 16330818 PMC2213324

[25] Chen C Liu Y Liu Y Zheng P. Mammalian target of rapamycin activation underlies HSC defects in autoimmune disease and inflammation in mice. J Clin Invest. (2010) 120:4091–101. 10.1172/JCI43873 20972332 PMC2964994

[26] de Bruin AM Demirel O Hooibrink B Brandts CH Nolte MA. Interferon-gamma impairs proliferation of hematopoietic stem cells in mice. Blood. (2013) 121:3578–85. 10.1182/blood-2012-05-432906 23487025

[27] Oduro KAJr Liu F Tan Q Kim CK Lubman O Fremont D. Myeloid skewing in murine autoimmune arthritis occurs in hematopoietic stem and primitive progenitor cells. Blood. (2012) 120:2203–13. 10.1182/blood-2011-11-391342 22855602 PMC3447779

[28] Snoeck HW Van Bockstaele DR Nys G Lenjou M Lardon F Haenen L Interferon gamma selectively inhibits very primitive CD342+CD38- and not more mature CD34+CD38+ human hematopoietic progenitor cells. J Exp Med. (1994) 180:1177–82. 10.1084/jem.180.3.1177 7520470 PMC2191667

[29] Zhang Y Harada A Bluethmann H Wang JB Nakao S Mukaida N. Tumor necrosis factor (TNF) is a physiologic regulator of hematopoietic progenitor cells: increase of early hematopoietic progenitor cells in TNF receptor p55-deficient mice *in vivo* and potent inhibition of progenitor cell proliferation by TNF alpha *in vitro*. Blood. (1995) 86:2930–7. 10.1182/blood.V86.8.2930.2930 7579385

[30] Esplin BL Shimazu T Welner RS Garrett KP Nie L Zhang Q. Chronic exposure to a TLR ligand injures hematopoietic stem cells. J Immunol. (2011) 186:5367–75. 10.4049/jimmunol.1003438 21441445 PMC3086167

[31] Takizawa H Fritsch K Kovtonyuk LV Saito Y Yakkala C Jacobs K. Pathogen-induced TLR4-TRIF innate immune signaling in hematopoietic stem cells promotes proliferation but reduces competitive fitness. Cell Stem Cell. (2017) 21:225–40.e5. 10.1016/j.stem.2017.06.013 28736216

[32] Takizawa H Regoes RR Boddupalli CS Bonhoeffer S Manz MG. Dynamic variation in cycling of hematopoietic stem cells in steady state and inflammation. J Exp Med. (2011) 208:273–84. 10.1084/jem.20101643 21300914 PMC3039863

[33] Klein SL Flanagan KL. Sex differences in immune responses. Nat Rev Immunol. (2016) 16:626–38. 10.1038/nri.2016.90 27546235

[34] Kwak HJ Liu P Bajrami B Xu Y Park SY Nombela-Arrieta C. Myeloid cell-derived reactive oxygen species externally regulate the proliferation of myeloid progenitors in emergency granulopoiesis. Immunity. (2015) 42:159–71. 10.1016/j.immuni.2014.12.017 25579427 PMC4303526

[35] Hu Y Smyth GK. ELDA: extreme limiting dilution analysis for comparing depleted and enriched populations in stem cell and other assays. J Immunol Methods. (2009) 347:70–8. 10.1016/j.jim.2009.06.008 19567251

[36] Laupland KB. Incidence of bloodstream infection: a review of population-based studies. Clin Microbiol Infect. (2013) 19:492–500. 10.1111/1469-0691.12144 23398633

[37] Cheers C Haigh AM Kelso A Metcalf D Stanley ER Young AM. Production of colony-stimulating factors (CSFs) during infection: separate determinations of macrophage-, granulocyte-, granulocyte-macrophage-, and multi-CSFs. Infect Immun. (1988) 56:247–51. 10.1128/IAI.56.1.247-251.1988 3257205 PMC259264

[38] Hirai H Zhang P Dayaram T Hetherington CJ Mizuno S Imanishi J. C/EBPbeta is required for 'emergency' granulopoiesis. Nat Immunol. (2006) 7:732–9. 10.1038/ni1354 16751774

[39] Walker F Zhang HH Matthews V Weinstock J Nice EC Ernst M. IL6/sIL6R complex contributes to emergency granulopoietic responses in G-CSF- and GM-CSF-deficient mice. Blood. (2008) 111:3978–85. 10.1182/blood-2007-10-119636 18156493

[40] Challen GA Boles N Lin KK Goodell MA. Mouse hematopoietic stem cell identification and analysis. Cytometry Part A. (2009) 75:14–24. 10.1002/cyto.a.20674 19023891 PMC2640229

[41] Morrison SJ Uchida N Weissman IL. The biology of hematopoietic stem cells. Annu Rev Cell Dev Biol. (1995) 11:35–71. 10.1146/annurev.cb.11.110195.000343 8689561

[42] Furusawa J Mizoguchi I Chiba Y Hisada M Kobayashi F Yoshida H. Promotion of expansion and differentiation of hematopoietic stem cells by interleukin-27 into myeloid progenitors to control infection in emergency myelopoiesis. PLoS Pathog. (2016) 12:e1005507. 10.1371/journal.ppat.1005507 26991425 PMC4798290

[43] Rashidi NM Scott MK Scherf N Krinner A Kalchschmidt JS Gounaris K. *In vivo* time-lapse imaging shows diverse niche engagement by quiescent and naturally activated hematopoietic stem cells. Blood. (2014) 124:79–83. 10.1182/blood-2013-10-534859 24850759 PMC4125355

[44] Rodriguez S Chora A Goumnerov B Mumaw C Goebel WS Fernandez L. Dysfunctional expansion of hematopoietic stem cells and block of myeloid differentiation in lethal sepsis. Blood. (2009) 114:4064–76. 10.1182/blood-2009-04-214916 19696201 PMC2774548

[45] Satake S Hirai H Hayashi Y Shime N Tamura A Yao H. C/EBPbeta is involved in the amplification of early granulocyte precursors during candidemia-induced “emergency” granulopoiesis. J Immunol. (2012) 189:4546–55. 10.4049/jimmunol.1103007 23024276

[46] Scumpia PO Kelly-Scumpia KM Delano MJ Weinstein JS Cuenca AG Al-Quran S. Cutting edge: bacterial infection induces hematopoietic stem and progenitor cell expansion in the absence of TLR signaling. J Immunol. (2010) 184:2247–51. 10.4049/jimmunol.0903652 20130216 PMC3837089

[47] Zhang H Nguyen-Jackson H Panopoulos AD Li HS Murray PJ Watowich SS. STAT3 controls myeloid progenitor growth during emergency granulopoiesis. Blood. (2010) 116:2462–71. 10.1182/blood-2009-12-259630 20581311 PMC2953883

[48] Dumont FJ Coker LZ. Interferon-alpha/beta enhances the expression of Ly-6 antigens on T cells *in vivo* and *in vitro*. Eur J Immunol. (1986) 16:735–40. 10.1002/eji.1830160704 3487457

[49] Malek TR Danis KM Codias EK. Tumor necrosis factor synergistically acts with IFN-gamma to regulate Ly-6A/E expression in T lymphocytes, thymocytes and bone marrow cells. J Immunol. (1989) 142:1929–36. 2493502

[50] Zhang P Nelson S Bagby GJ Siggins R II Shellito JE Welsh DA. The lineage-c-Kit+Sca-1+ cell response to *Escherichia coli* bacteremia in Balb/c mice. Stem Cells. (2008) 26:1778–86. 10.1634/stemcells.2007-1027 18483422 PMC2731662

[51] Kiel MJ Yilmaz OH Iwashita T Yilmaz OH Terhorst C Morrison SJ. SLAM family receptors distinguish hematopoietic stem and progenitor cells and reveal endothelial niches for stem cells. Cell. (2005) 121:1109–21. 10.1016/j.cell.2005.05.026 15989959

[52] Morita Y Ema H Nakauchi H. Heterogeneity and hierarchy within the most primitive hematopoietic stem cell compartment. J Exp Med. (2010) 207:1173–82. 10.1084/jem.20091318 20421392 PMC2882827

[53] Osawa M Hanada K Hamada H Nakauchi H. Long-term lymphohematopoietic reconstitution by a single CD34-low/negative hematopoietic stem cell. Science. (1996) 273:242–5. 10.1126/science.273.5272.242 8662508

[54] Pietras EM Reynaud D Kang YA Carlin D Calero-Nieto FJ Leavitt AD. Functionally distinct subsets of lineage-biased multipotent progenitors control blood production in normal and regenerative conditions. Cell Stem Cell. (2015) 17:35–46. 10.1016/j.stem.2015.05.003 26095048 PMC4542150

[55] Manz MG Boettcher S. Emergency granulopoiesis. Nat Rev Immunol. (2014) 14:302–14. 10.1038/nri3660 24751955

[56] Ueda Y Kondo M Kelsoe G. Inflammation and the reciprocal production of granulocytes and lymphocytes in bone marrow. J Exp Med. (2005) 201:1771–80. 10.1084/jem.20041419 15939792 PMC1952536

[57] Burberry A Zeng MY Ding L Wicks I Inohara N Morrison SJ. Infection mobilizes hematopoietic stem cells through cooperative NOD-like receptor and Toll-like receptor signaling. Cell Host Microbe. (2014) 15:779–91. 10.1016/j.chom.2014.05.004 24882704 PMC4085166

[58] Mullally A Bruedigam C Poveromo L Heidel FH Purdon A Vu T. Depletion of Jak2V617F myeloproliferative neoplasm-propagating stem cells by interferon-alpha in a murine model of polycythemia vera. Blood. (2013) 121:3692–702. 10.1182/blood-2012-05-432989 23487027 PMC3643767

[59] Maltby S Hansbro NG Tay HL Stewart J Plank M Donges B. Production and differentiation of myeloid cells driven by proinflammatory cytokines in response to acute pneumovirus infection in mice. J Immunol. (2014) 193:4072–82. 10.4049/jimmunol.1400669 25200951 PMC4185243

[60] Pascutti MF Erkelens MN Nolte MA. Impact of viral infections on hematopoiesis: from beneficial to detrimental effects on bone marrow output. Front Immunol. (2016) 7:364. 10.3389/fimmu.2016.00364 27695457 PMC5025449

[61] Zhao JL Ma C O'Connell RM Mehta A DiLoreto R Heath JR. Conversion of danger signals into cytokine signals by hematopoietic stem and progenitor cells for regulation of stress-induced hematopoiesis. Cell Stem Cell. (2014) 14:445–59. 10.1016/j.stem.2014.01.007 24561084 PMC4119790

[62] Orford KW Scadden DT. Deconstructing stem cell self-renewal: genetic insights into cell-cycle regulation. Nat Rev Genet. (2008) 9:115–28. 10.1038/nrg2269 18202695

[63] Kim JM Oh YK Kim YJ Youn J Ahn MJ. *Escherichia coli* up-regulates proinflammatory cytokine expression in granulocyte/macrophage lineages of CD34 stem cells via p50 homodimeric NF-kappaB. Clin Exp Immunol. (2004) 137:341–50. 10.1111/j.1365-2249.2004.02542.x 15270851 PMC1809125

[64] Cottler-Fox MH Lapidot T Petit I Kollet O DiPersio JF Link D Stem cell mobilization. Hematol Am Soc Hematol Educ Program. (2003) 419–37. 10.1182/asheducation-2003.1.419 14633793

[65] Greenbaum AM Link DC. Mechanisms of G-CSF-mediated hematopoietic stem and progenitor mobilization. Leukemia. (2011) 25:211–7. 10.1038/leu.2010.248 21079612

[66] Glait-Santar C Gur-Cohen S Canaani J Kollet O Lapidot T Egress and mobilization of hematopoietic stem and progenitor cells: a dynamic multi-facet process. In: Cowan CA editor. StemBook. Cambridge, MA: IOS Press (2008). p. 1–37. 10.3824/stembook.1.39.1 23658994

[67] Calvi LM Link DC. The hematopoietic stem cell niche in homeostasis and disease. Blood. (2015) 126:2443–51. 10.1182/blood-2015-07-533588 26468230 PMC4661168

[68] Crane GM Jeffery E Morrison SJ. Adult haematopoietic stem cell niches. Nat Rev Immunol. (2017) 17:573–90. 10.1038/nri.2017.53 28604734

[69] Scadden DT. Nice neighborhood: emerging concepts of the stem cell niche. Cell. (2014) 157:41–50. 10.1016/j.cell.2014.02.013 24679525 PMC4161226

[70] Wilson A Trumpp A. Bone-marrow haematopoietic-stem-cell niches. Nat Rev Immunol. (2006) 6:93–106. 10.1038/nri1779 16491134

[71] Baldridge MT King KY Goodell MA. Inflammatory signals regulate hematopoietic stem cells. Trends Immunol. (2011) 32:57–65. 10.1016/j.it.2010.12.003 21233016 PMC3042730

