# Supplementary material for: Bacteria-Induced Acute Inflammation Does Not Reduce the Long-Term Reconstitution Capacity of Bone Marrow Hematopoietic Stem Cells
Source: Front Immunol. 2020 Apr 16;11:626. doi: 10.3389/fimmu.2020.00626 (PMC7179742; doi:10.3389/fimmu.2020.00626)
Supplement: Supplementary file 1 [file Data_Sheet_1.pdf]

frontiers

Supplementary Material

Table S1. Raw data of the limiting dilution assay. Experiments were conducted as described in **Figure 2D**. Shown are the number of populations tested and the number of assays giving positive transplantation (≥0.5% engraftment).

Dose	Number of populations tested	Number of positive populations	Group
250	5	4	PBS
500	3	3	PBS
1000	4	4	PBS
250	5	2	HIEC
500	5	2	HIEC
1000	5	3	HIEC
4000	5	5	HIEC
8000	5	5	HIEC

Table S2. Calculation of the percentage of functional HSCs in the bone marrow mononuclear cells (BMMCs) in PBS-treated and HIEC-challenged mice. LSK cell percentage in BMMCs (mean ± SD) was calculated as described in **Figure 1C**. HSC frequency in LSK cells was calculated as described in **Figure 2D-F**. Percentage of HSCs in BMMCs (mean ± SD) was calculated accordingly.

Group	LSK cell percentage in BMMC (%) (mean ± SD)	HSC frequency in LSK cells	Percentage of HSC in BMMC (%) (mean ± SD)
PBS	0.14 ± 0.03%	1/140	$9.67 \pm 1.85 \times 10^{-4}$
HIEC	1.00 ± 0.08%	1/852	$11.75 \pm 0.98 \times 10^{-4}$

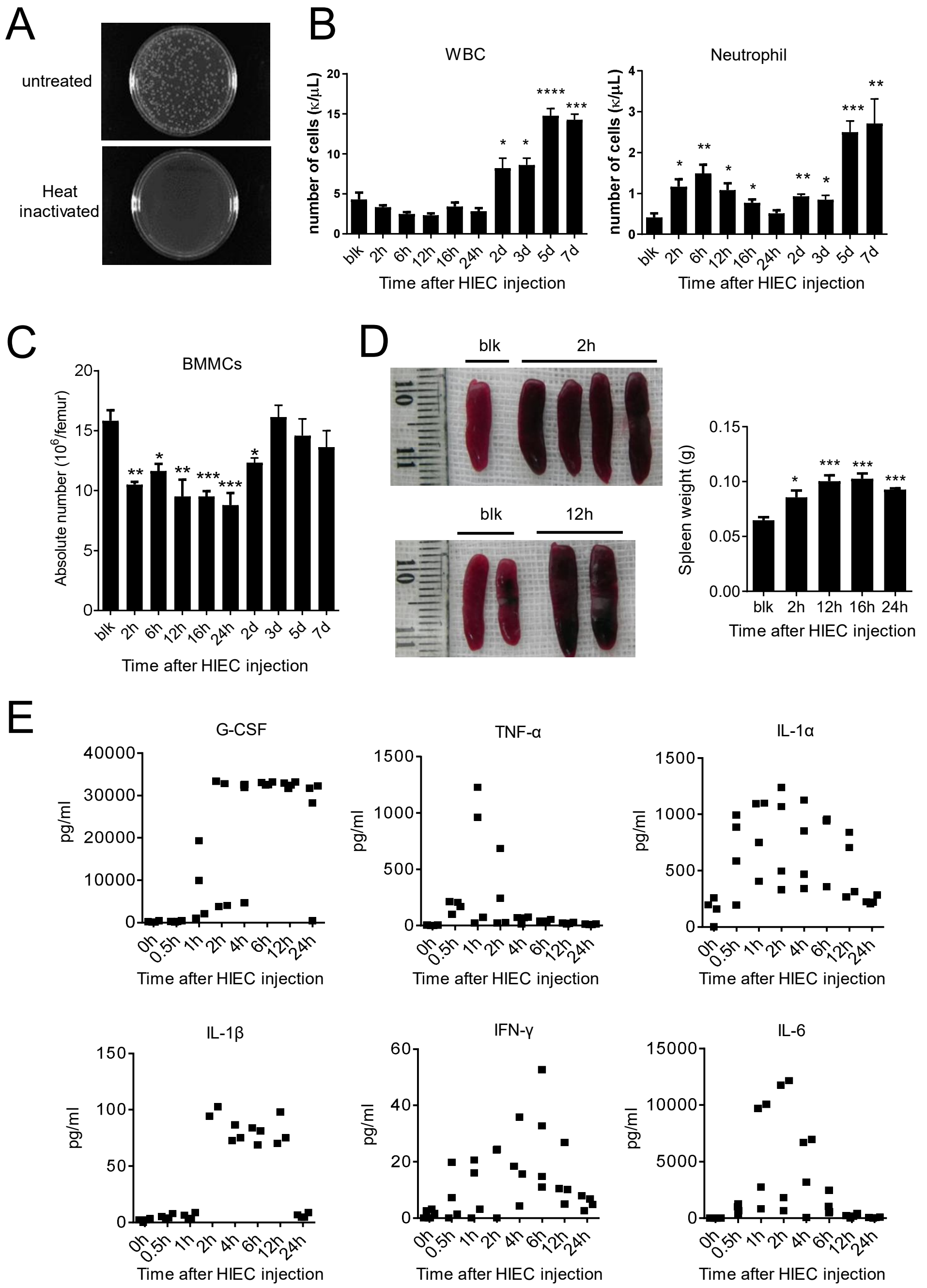

Figure S1. (A) Heat inactivated *E. coli* (HIEC) lost proliferation capability. (B) Absolute number of WBCs and neutrophils in the peripheral blood at different time points after HIEC challenge. Data shown are means ± SD (n = 5 mice). $*p < 0.05$, $**p < 0.01$, $***p < 0.001$, $****p < 0.0001$ versus untreated mice (blk). (C) Absolute number of BM mononuclear cells at different time points after HIEC challenge. Data shown are means ± SD (n = 5 mice). $*p < 0.05$, $**p < 0.01$, $***p < 0.001$, $****p < 0.0001$ versus untreated mice (blk). (D) HIEC-elicited acute infection induces splenomegaly. Data shown are means ± SD (n = 5 mice). $*p < 0.05$, $**p < 0.01$, $***p < 0.001$, $****p < 0.0001$ versus untreated mice (blk). (E) Cytokine production in HIEC-challenged mice. The levels of indicated cytokines in the serum were measured at each indicated time points. Four mice were used for each data point.

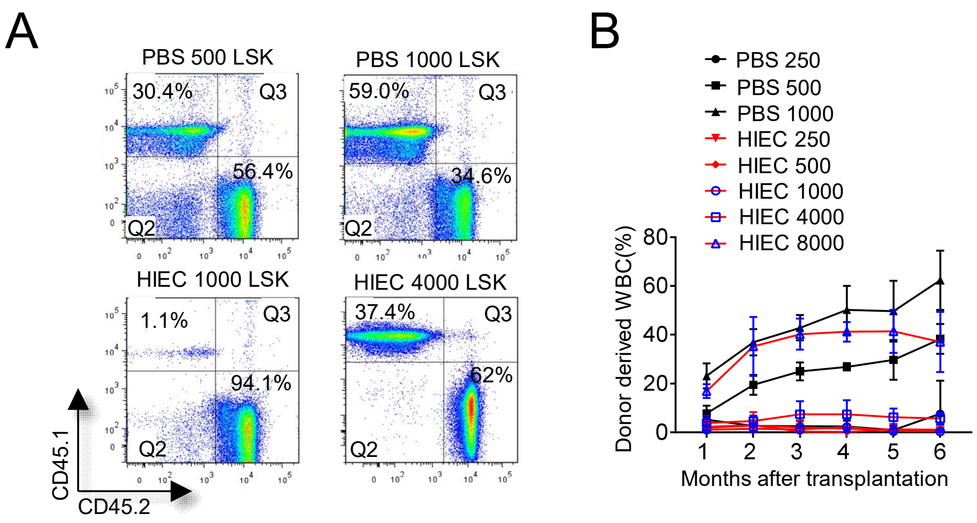

Figure S2. Limiting dilution analyses (LDA) to assess the frequency of functional HSCs (competitive repopulating units, or CRU) in LSK cell population. The experiment was conducted as described in Figure 2D. (A) Representative FACS plots showing the percentage of $CD45.1^+$ donor-derived WBCs in the peripheral blood six months after transplantation. (B) The percentage of $CD45.1^+$ donor-derived WBCs in the peripheral blood at the indicated time points. Data shown are means ± SD of n = 5 mice.

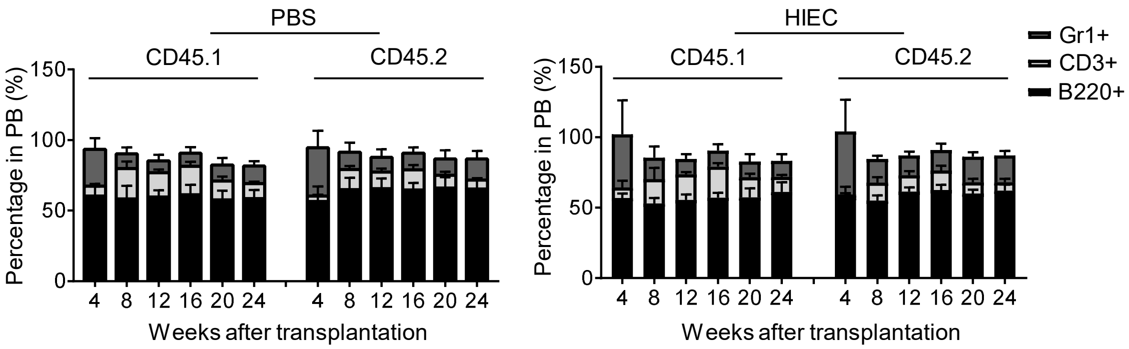

Figure S3. The percentage of donor-derived CD45.2^{+} and CD45.1^{+} cells in indicated blood lineages in the PB of primary recipients at the indicated time points. The experiment was conducted as described in **Figure 3**. Data shown are mean ± SD of n = 5 mice.

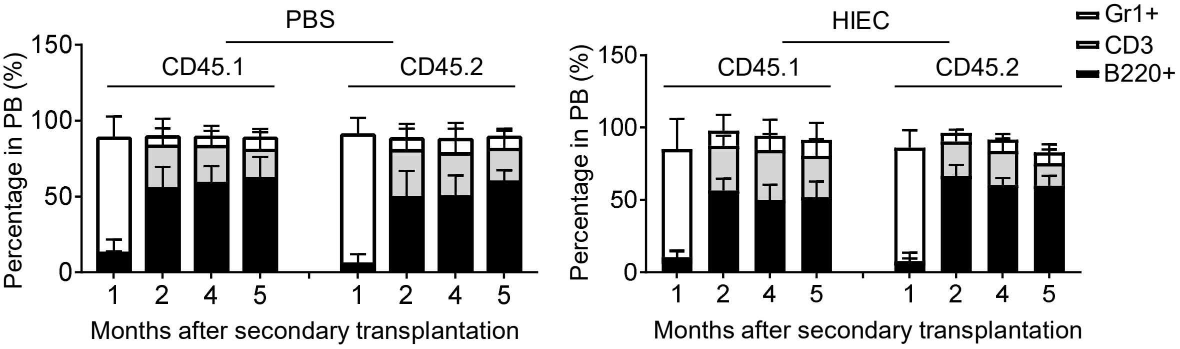

Figure S4. The percentage of donor-derived CD45.2$^+$ and CD45.1$^+$ cells in the indicated blood lineages in the PB of the secondary recipients at the indicated time points. The experiment was conducted as described in **Figure 4**. Data shown are mean ± SD of n = 5 mice.
